# Isoform Remodeling and Promoter Switching Identify Altered Transcriptional Regulation in Alzheimer's Disease Frontal Cortex

**DOI:** 10.21203/rs.3.rs-10820900/v1

**Published:** 2026-09-13

**Authors:** Jelard Aquino, Alice Lee, Jennifer Zheng, Wai Lim Ku, Mira Han, Jingchun Chen

**Affiliations:** University of Nevada; University of Nevada; University of Nevada; Howard University; University of Nevada; University of Nevada

**Keywords:** Alzheimer’s disease, long-read RNA-seq, promoter switching, differential isoform usage, alternative splicing, isoform dysregulation

## Abstract

**BACKGROUND:**

Alzheimer’s disease (AD) is associated with transcriptional dysregulation, but gene-level analyses may overlook isoform changes. We investigated how differential isoform usage, promoter selection, and splicing contribute to transcriptome remodeling in the AD frontal cortex.

**METHODS:**

We analyzed Oxford Nanopore long-read RNA sequencing data from frontal cortex of six individuals with AD and six controls. Transcripts were quantified using Bambu, and differential isoform usage was assessed using IsoformSwitchAnalyzeR. Promoter usage and SUPPA2-derived percent-spliced-in values were tested using beta regression. Transcription factor motif enrichment was evaluated at differentially used promoters. Transcript-level effects were compared with an independent short-read cohort.

**RESULTS:**

We identified 109 isoform-switching events involving 96 isoforms across 86 genes, including *CLSTN3*, *SNCA*, *BECN1*, *USP10*, and *EGFR*. Several switches altered predicted nonsense-mediated decay sensitivity. Eleven genes exhibited promoter switching, comprising 46 differentially used promoters: 31 with increased and 15 with decreased usage in AD. These promoters were enriched for 179 TF motifs. IRF7, IRF8, IRF9, and STAT1::STAT2 motifs were top-ranked among promoters with increased usage, whereas NFKB1 and NFKB2 motifs were top-ranked among those with decreased usage. Targeted analysis identified 14 significant differential splicing events with sex-associated effects, including *BECN1*. Although cross-platform concordance was weak, 16 of 26 transcripts with large effects in both datasets changed concordantly, and 13 showed large, concordant effects of similar magnitude.

**CONCLUSIONS:**

AD-associated transcriptome remodeling extends beyond gene-level abundance to include changes in isoform proportions, promoter selection, and transcript structure. These findings implicate transcription initiation and RNA processing as complementary layers of dysregulation in AD.

## 1. BACKGROUND

Alzheimer’s disease (AD) is characterized by progressive neurodegeneration and widespread alterations in gene expression throughout affected brain regions. Numerous bulk and single-cell transcriptomic studies have identified large-scale transcriptional dysregulation in AD, implicating synaptic signaling, immune activation, mitochondrial pathways, and myelination, but most have emphasized total gene-level changes rather than transcript-level complexity[1-5]. This gene-level view can miss the regulated usage of distinct transcript isoforms, which is a major source of molecular diversity in the brain.

Alternative transcript usage, including isoform switching and alternative promoter usage, expands the functional repertoire of the genome and mediates precise spatial and temporal control of neuronal gene expression. Through changes in 5’ untranslated regions, coding sequences, and regulatory elements, alternative promoters can influence translation efficiency, protein localization, and stability, often independent of changes in overall gene expression. In the brain, activity-dependent promoter usage and other RNA processing mechanisms have been linked to synaptic plasticity, learning, and memory, highlighting promoter-level regulation as a key modulator of neuronal function[6-9].

Emerging evidence suggests that promoter switching is perturbed in AD and related tauopathies. At the *MAPT* gene, for example, an alternative promoter activation locus generates shorter tau mRNA transcripts that are enriched in AD and progressive supranuclear palsy brains, demonstrating that pathological promoter switching can remodel the transcript architecture of a key neurodegenerative disease gene[10]. Transcriptome-wide studies in AD also reported altered promoter usage and transcription start site (TSS) selection in genes such as *APOE*, implying that promoter-level regulation contributes more broadly to disease-associated isoform diversity[8].

More generally, isoform-level dysregulation is increasingly recognized as a prominent feature of AD, with many genes exhibiting differential isoform usage (DIU) despite minimal or no detectable gene-level differential expression[8, 11-13]. DIU analyses in AD brain have identified isoform switches in risk and causal genes such as *APP* and *BIN1*[8], as well as widespread changes in splicing-related pathways that track with disease stage and pathology burden. Yet, most prior work has emphasized alternative splicing (AS) and 3’ end processing, leaving the contribution of alternative promoter usage and promoter switching to AD-associated isoform changes underexplored[8, 11-13].

A key unresolved question in AD research is which transcription factors (TFs) and regulatory networks orchestrate promoter-level changes. Evidence from microglial and type-I interferon studies points to interferon regulatory factors, especially IRF7[14] and IRF8[15], as well as NF- *κ* B family[16] signaling, as key upstream regulators of disease-associated inflammatory programs. Defining the TF motifs enriched at promoters undergoing AD-associated switching could therefore provide mechanistic insight into the upstream regulatory programs that drive transcriptomic reprogramming in neurodegeneration.

To investigate DIU and promoter switching, we applied an integrative promoter-centric transcriptomic framework to analyze publicly available long-read RNA-seq data from the postmortem frontal cortex of individuals with AD and controls. Additionally, alternative splicing events were examined in an independent bulk RNA-seq frontal cortex dataset to assess replication of isoform-level alterations. By linking isoform-level changes to promoter-specific regulation and putative TF drivers, this study clarifies how alternative promoter usage contributes to transcriptome remodeling in AD and highlights candidate regulatory factors associated with disease-related promoter activities.

## 2. METHODS

### 2.1 Long-read RNA-seq data Processing

Oxford Nanopore Technologies (ONT) long-read RNA-seq data were obtained from Heberle *et al.*[17] (syn52047893, SRP456327) and consisted of 12 postmortem frontal cortex samples, including 6 Alzheimer’s disease (AD) and 6 controls (CT). Additional details about the data are available in Heberle *et al.*[17] Raw FASTQ files were downloaded and preprocessed following the pipeline described by Heberle *et al.*[17] Reads were filtered and oriented using Pychopper[18] v2.7.2, aligned to the GRCh38 reference genome (without alternative contigs and with added ERCC Sequences) using minimap2[19], and filtered to remove alignments with MAPQ < 10 using SAMtools[20] v1.6 (Fig. 1B).

To increase sensitivity for detecting lowly expressed and novel isoforms, all 12 aligned BAM files were merged before transcript discovery with Bambu[21] v1.0.0, instead of performing transcript discovery separately for each sample as described in Heberle *et al.*[17] This joint transcript discovery approach produced an expanded Bambu transcript annotation containing additional transcripts relative to the original sample-by-sample analysis.

Transcript counts were converted to transcripts per million (TPM) to account for both transcript length and sequencing depth. First, transcript lengths were converted from base pairs to kilobases. Raw counts for each transcript from Bambu were then divided by the corresponding transcript length in kilobases to obtain length-normalized expression rates (reads per kilobase). Next, these rates were summed within each sample to estimate the total transcriptional output. Finally, each transcript’s rate was divided by the sample-specific sum and multiplied by one million, producing TPM values that represent the relative abundance of each transcript within a sample. TPM is defined as follows:

$$TPM_{i,s}=\frac{C_{i,s}∕(L_{i}∕1000)}{{\sum \;}_{j=1}^{N}C_{j,s}∕(L_{i}∕1000)}\times 10^{6}$$

where $C_{i,s}$ is the raw Bambu count for transcript i in sample s, $L_{i}$ is transcript length in base pairs, and N is the total number of quantified transcripts. These TPM values were used in downstream processing, including the promoter switching analysis and alternative splicing event-level analysis.

### 2.2 Differential Isoform Usage (DIU) Analysis

Transcript counts generated by Bambu were analyzed using IsoformSwitchAnalyzeR[22] v2.8.0 to assess differential isoform usage (DIU) between AD and CT individuals. Heberle *et al.* prioritized novel isoform detection without examining DIU; here, we assess whether isoform usage is associated with AD. Statistical testing for DIU was performed using *isoformSwitchTestDEXSeq*, which models transcript proportions within genes using DEXSeq’s[23] negative binomial model. *P*-values were corrected for multiple testing using the Benjamini-Hochberg procedure, and genes were considered to show significant changes in isoform usage at an adjusted *p* ≤ 0.05 and dIF > 10%. Isoform coding potential was assessed within IsoformSwitchAnalyzeR, and isoforms were classified as predicted NMD-sensitive when the stop codon was located more than 50 nucleotides upstream of the final exon-exon junction (premature termination codon, PTC). Isoforms for which an open reading frame could not be assigned were treated as indeterminate and excluded from NMD summaries.

### 2.3 Promoter Switching Analysis

Promoter regions were defined for each Bambu-identified transcript as the interval from 1,000 base pairs (bp) upstream to 100 bp downstream of each transcript's TSS. Because transcript abundance is quantified at the transcript level, promoter-level expression was derived by aggregating the expression of transcripts initiating within each promoter region.

Specifically, promoter-level TPM ($TPM_{p,s}$) was defined as the sum of TPM values of all transcripts whose TSS falls within promoter p in sample s:

$$TPM_{p,s}=\sum_{\;t\in T(p)}TPM_{t,s}$$

where T(p) denotes the set of transcripts assigned to promoter p.

Promoter usage was then calculated as the relative contribution of each promoter to the total expression of its corresponding gene:

$$PromoterUsage_{p,s}=\frac{TPM_{p,s}}{{\sum \;}_{p^{\prime }\in G(p)}TPM_{p^{\prime }\;,s}}$$

where p denotes promoters, s denotes samples, and G(p) represents the set of promoters belonging to a gene containing promoter p.

Differential promoter usage between AD and CT individuals was assessed using beta regression, which is appropriate for modeling continuous proportions constrained to the open interval (0,1). For each promoter, promoter usage values were modeled as:

$$PromoterUsage_{p,s}=Beta(\mu_{p,s},\phi \;)$$

with the mean parameter $\mu_{p,s}$ linked to the experimental condition via a logit link function:

$$logit\left(\mu_{p,s}\right)=\beta_{0}+\beta_{1}\cdot Condition_{s}$$

where $Condition_{s}$ indicates AD or CT status, $\beta_{0}$ represents the baseline promoter usage, and $\beta_{1}$ corresponds to the logit fold change (log-odds ratio) in promoter usage between AD and CT individuals. Statistical significance of differential promoter usage was assessed using Wald tests on $\beta_{1}$. *P*-values were adjusted using the Benjamini-Hochberg procedure, with events considered significant at an adjusted *p* ≤ 0.05 and absolute $log_{2}\;(fold\;change)\geq 1$.

Promoter switching was defined at the gene level as the presence of at least two promoters with significant differential usage in opposite directions (i.e., one promoter with $\beta_{1}>0$ while another with $\beta_{1}<0$), consistent with a redistribution of promoter usage within the same gene.

### 2.4 Splicing Graph-Based Visualization of Transcript Expression

Transcript, promoter, and splice junction expressions were visualized using the splicing graph framework described by Aquino *et al.*[24], in which exons are represented as edges and splice junctions as nodes. Splice junction counts were obtained using RegTools[25] v1.0.0 from aligned BAM files, while transcript-level counts and promoter counts were derived from Bambu. Splicing graphs were generated separately by gene, showcasing a direct comparison of transcript and promoter expression and AS patterns between AD and CT conditions.

### 2.5 Transcription Factor (TF)/Motif Enrichment Analysis

Promoters exhibiting significant switching between AD and CT individuals (adjusted *p* ≤ 0.05) were retained for TF motif enrichment analysis. Promoter genomic coordinates and strand information were converted into GRanges objects using the GenomicRanges[26] v1.6.0 package. Promoters were stratified by direction of change, with increased and decreased promoter usage in AD analyzed separately.

TSSs were defined in a strand-aware manner, using the 5’ coordinate for plus-strand promoters and the 3’ coordinate for minus-strand promoters. Although promoter usage was quantified using narrower −1kb/+100 bp promoter intervals, motif enrichment was performed using broader strand-aware ± 2 kb windows centered on each promoter TSS to capture a wider TSS-proximal regulatory sequence context. These ± 2 kb motif-scanning windows were generated using GenomicFeatures, and the corresponding DNA sequences were extracted from the human reference genome (GRCh38) using BSgenome.Hsapiens.UCSC.hg38[27] v1.4.5. The extracted promoter-centered sequences were then used as input for transcription factor motif enrichment analysis.

TF motif enrichment was performed using PWMEnrich[28] v4.44.0 with a curated collection of human position weight matrices (PWMLogn.hg19.MotifDb.Hsap[29]). Motif enrichment was assessed by comparing observed motif affinity scores within promoter sequences to a precomputed log-normal background model, yielding motif-specific enrichment scores and p-values. Motifs belonging to the same TF family were summarized using the groupReport function. Enrichment results were ranked by statistical significance and visualized according to motif enrichment score and *p*-value. Enriched motifs corresponding to TFs previously implicated in AD were highlighted using a curated list of AD-associated TFs.

### 2.6 Alternative Splicing Event-level Analysis

#### 2.6.1 Targeted Event-level Analysis of Differential Isoform Usage Genes

Alternative splicing event-level analysis was performed as a targeted follow-up to the differential isoform usage analysis. Rather than conducting a genome-wide differential splicing screen, this analysis was restricted to genes with significant differential isoform usage between AD and CT samples. This approach was used to characterize the specific transcript-structure events contributing to isoform proportion changes among genes already identified as isoform-dysregulated in AD.

Transcripts assigned to the genes with significant differential isoform usage were extracted from the Bambu-derived transcript annotation and used to match the events generated by SUPPA2[30] v2.3. Alternative splicing events were defined and quantified for seven event classes: skipped exon (SE), mutually exclusive exon (MX), retained intron (RI), alternative 5’ splice site (A5), alternative 3’ splice site (A3), alternative first exon (AF), and alternative last exon (AL). Transcript-level TPM values derived from Bambu were used to calculate percent spliced-in (PSI) values for each event.

SUPPA2 defines PSI as the fraction of transcript expression supporting inclusion of a given splicing event.

For an AS event e, PSI is defined as:

$$PSI_{e}=\frac{{\sum \;}_{a\in \;I_{e}}TPM_{a}}{{\sum \;}_{a\in \;I_{e}}TPM_{a}+{\sum \;}_{b\in \;E_{e}}TPM_{b}}$$

where $I_{e}$ denotes transcripts that include the event, $E_{e}$ denotes transcripts that exclude the event, and TPM values are derived from transcript-level quantification.

#### 2.6.2 Beta Regression Modeling of PSI

Differential splicing between AD and CT individuals was tested using beta regression, which is appropriate for modeling continuous outcomes bounded in (0, 1) and naturally accommodates mean–variance coupling. Because beta regression requires response values to lie within the open interval (0,1), PSI values were transformed prior to model fitting using

$$\frac{\psi \;(n-1)+0.5}{n}$$

where n represents the number of non-missing PSI observations for a given event. Disease status was modeled as the primary predictor, and sex was included as a covariate.

For each alternative splicing event, PSI values were modeled as:

$$\psi_{i}=Beta(\mu_{i}\phi \;,(1-\mu_{i})\;\phi )\;\;logit\;(\mu_{i})=\beta_{0}+\beta_{1}\cdot group_{i}+\beta_{2}\cdot sex_{i}$$

where $\mu_{i}$ is the expected PSI for sample i, ϕ is a precision parameter capturing the between-sample variability, and $\beta_{1}$ captures the effect of disease status on splicing inclusion. To test for disease-associated PSI differences, the full model was compared with a reduced model that excluded disease status but retained sex as a covariate:

$$logit\;(\mu_{i})=\beta_{0}+\beta_{2}\cdot sex_{i}$$

As an exploratory analysis, we also tested whether PSI variation among significant events was better explained by a model allowing disease-associated effects to vary by sex. For each event, a full interaction model including disease status, sex, and a condition-by-sex interaction term was fit as:

$$logit\;(\mu_{i})=\beta_{0}+\beta_{1}\cdot group_{i}\cdot \beta_{2}\cdot sex_{i}$$

compared with a reduced model containing sex alone:

$$logit\;(\mu_{i})=\beta_{0}+\beta_{2}\cdot sex_{i}$$

Statistical significance was assessed using a likelihood ratio test comparing the full and reduced models. The likelihood ratio statistic was calculated as 2 * (Log-Likelihood of Full Model – Log-Likelihood of Reduced Model) and evaluated against a chi-square distribution using the difference in model degrees of freedom. P-values were adjusted using the Benjamini-Hochberg procedure, with events considered significant at adjusted *p* ≤ 0.05 and dPSI ≥ 0.10.

Although PSI values were computed using SUPPA2, differential splicing was not assessed using SUPPA2’s default statistical framework. SUPPA2 relies on an empirical abundance-stratified null distribution of Δ PSI values derived from replicate differences, which does not explicitly model PSI as a bounded response variable and does not allow straightforward inclusion of covariates. In contrast, beta regression directly models PSI as a continuous variable constrained to the unit interval, enables explicit modeling of covariates such as sex, and provides coefficient-based statistical inference for disease-associated splicing changes.

### 2.7 Independent Short-read RNA-seq Concordance Analysis

We compared transcript-level TPM estimates from the long-read dataset with an independent short-read bulk RNA-seq dataset from postmortem prefrontal cortex (European Nucleotide Archive: ERP161086[31]), consisting of 150-bp Illumina paired-end reads from AD (n = 7) and CT (n = 7) individuals. Reads were aligned to the GRCh38 reference genome, and TPM was quantified using Salmon[32] v0.14.2. Analyses were restricted to transcripts assigned to the significant gene set and present in both the long-read and short-read TPM matrices. TPM values were log-transformed as log2(TPM + 1). For each shared transcript, mean expression was calculated separately in AD and CT samples, and an AD-vs-CT effect size was computed as:

$$\Delta_{\;AD-CT}=mean\;log_{2}{\left(TPM+1\right)}_{AD}-mean\;log_{2}{\left(TPM+1\right)}_{CT}$$

This produced a long-read effect size and short-read effect size for each transcript. Transcript-level concordance between platforms was evaluated by comparing the direction and magnitude of these AD-vs-CT effects. Transcripts were considered directionally concordant if the long-read and short-read effect sizes had the same sign, indicating that both platforms showed higher expression in AD or lower expression in AD relative to CT. Transcripts were prioritized if they showed a large AD-vs-CT difference in both platforms, defined as an absolute log2 effect size above the selected threshold in both long and short-read data. To avoid prioritizing transcripts with low or absent expression, transcripts were also required to meet a minimum TPM expression threshold in at least one platform. Similarity in effect magnitude was quantified using an effect-size ratio, calculated as the smaller absolute effect size divided by the larger absolute effect size:

$$effect\;size\;ratio=\frac{min(|\Delta_{\;LR}|,\;|\Delta_{\;SR}|)}{max(|\Delta_{\;LR}|,\;|\Delta_{\;SR}|)}$$

Values closer to 1 indicate more similar AD-vs-CT effect magnitudes between platforms. Transcripts were classified as having large, similar AD-vs-CT differences if they were expressed above the minimum TPM threshold, showed large absolute AD-vs-CT effects in both platforms, changed in the same direction, and exceeded the minimum effect-size ratio threshold.

### 2.8 Statistics

All statistical methods and tests used in this paper are described in the figure legends, Methods, Supplementary Figures, or main text as appropriate.

## 3. RESULTS

Publicly available ONT long-read RNA-seq data from postmortem brain samples of CT (n = 6) and AD (n = 6) individuals were analyzed in this study (Fig. 1). Full-length transcript models derived from long-read sequencing enabled a unified framework for examining transcriptional regulation at multiple levels, allowing us to evaluate AD-associated changes in (1) isoform usage (within-gene shifts in transcript proportions), (2) promoter usage (within-gene shifts in TSS selection), and (3) TF/motif enrichment analysis. We further performed (4) AS analysis at the event level to get a deeper understanding of how splicing regulates these mechanisms. Together, these analyses provide a comprehensive perspective on AD-associated transcriptional regulation across isoform structure, promoter usage, TF activities, and splicing events.

### 3.1 Quality Control and Principal Component Analysis (PCA) Comparison from Heberle *et al.*

All basic sequencing metrics, including reads at each filtering level, median read length, full-length counts, and unique counts, were highly consistent with those previously reported by Heberle *et al.*[17] (**Supplementary Fig. 1**), confirming the reproducibility and reliability of the dataset. Principal component analysis (PCA) of TPM-normalized transcript counts showed partial separation between AD and CT individuals along the first two components, although three CT samples clustered with the AD group, indicating modest heterogeneity within the CT cohort (Fig. 2A). This separation is consistent with disease-associated transcriptomic differences and aligns with previous reports of differential RNA isoform expression by Heberle *et al.*[17]

### 3.2 Differential Isoform Usage Identifies AD-associated Remodeling

We first asked whether AD was associated with changes in transcript isoform proportions within genes. Differential isoform usage analysis identified 109 significant isoform-switching events involving 96 isoforms across 86 genes at FDR < 0.05 and ∣dIF∣ > 10% (Fig. 2B-C; **Supplementary Table 1**). These results indicate that AD-associated transcriptomic dysregulation extends beyond changes in total gene expression and includes remodeling of isoform composition within individual genes.

Representative isoform-switching events are shown for *DMXL1* and *CLSTN3*, selected to show two distinct consequences of isoform redistribution. In *DMXL1*, the significant switch involved increased usage of a nonsense-mediated decay (NMD)-sensitive transcript in AD, accompanied by higher total gene expression and higher expression of the switching isoforms in AD (Fig. 2D). This combination exemplifies why gene-level quantification alone can be misleading: although *DMXL1* appears upregulated in AD, a greater proportion of its transcriptional output is directed toward an isoform predicted to be degraded, so the apparent increase does not correspond to increased functional protein. *DMXL1* protein has been identified among the most predictive brain proteins distinguishing asymptomatic AD from cognitively normal controls in a machine-learning analysis of deep proteomic data from six cohorts[33], suggesting that *DMXL1* dysregulation may arise early in the disease process. PTC status could be assigned for 93 of the 96 switching isoforms, of which 15 were predicted NMD-sensitive: 3 with increased usage in AD, including the *DMXL1* isoform, and 6 with increased usage in controls. The remaining PTC-positive isoforms belonged to long non-coding genes, for which NMD prediction is less readily interpretable (**Supplementary Table 1**). In *CLSTN3*, two isoforms exhibited reciprocal switching, with CLSTN3-01 showing higher usage in controls and CLSTN3-02 showing higher usage in AD (Fig. 2E). *CLSTN3* encodes calsyntenin-3 (alcadein β), which is upregulated by Aβ in cortical neurons and accumulates in dystrophic neurites surrounding amyloid plaques[34, 35], and which is a γ-secretase substrate whose cleavage product, p3-Alcβ, restores the viability of neurons impaired by Aβ42 toxicity[36]. Therefore, a shift in the isoform composition is relevant to local synaptic pathology. Together, these samples demonstrate how AD-associated isoform dysregulation can involve both changes in productive transcript isoforms and shifts toward transcripts with predicted regulatory or decay-associated consequences.

### 3.3 Promoter Switching Contributes to a Subset of AD-associated Isoform Changes

We next tested whether altered transcription start site selection contributed to the DIU signal. Promoter usage was quantified by aggregating transcript TPM values according to shared TSS-proximal promoter regions and modeling promoter proportions within each gene. Among the 86 genes with significant DIU, 11 exhibited promoter switching, defined as reciprocal differential usage of at least two promoters within the same gene (**Supplementary** Fig. 2; **Supplementary Table 2**). At the promoter level, we identified 46 differentially used promoters, including 31 with increased usage and 15 with decreased usage in AD (Fig. 3A).

Three promoter switching genes with prior links to AD-relevant biology are highlighted as representative examples, with promoter-level usage differences shown in Fig. 3B. *SNCA* encodes -synuclein, which accumulates as Lewy-body co-pathology in a substantial fraction of AD brains and has been associated with Lewy-body burden in AD[37, 38], making a redistribution of its transcription initiation of potential pathological relevance. *NOTCH4* provides the simplest illustration of promoter switching: its two differentially used promoters lie approximately 7 kb apart and are each linked to a single transcript, so the redistribution of promoter usage connects directly onto a change in transcript composition without contributions from other transcripts at the locus. *NOTCH4* also lies within the chromosome 6p21 MHC region, where variants have been associated with dementia[39, 40]. *NSD2* was selected to show the structural consequences of promoter switching rather than for prior disease association: its two promoters lie approximately 35 kb apart and show the clearest separation between transcription initiation sites among the switching genes, allowing the downstream effect on transcript structure to be traced directly (Fig. 3C). Together, these findings indicate that alternative transcription initiation contributes to a subset of AD-associated isoform changes and suggest that promoter-level regulation may reshape transcript composition in AD cortex.

The splicing graph of NSD2 further illustrates how promoter switching can propagate through downstream transcript structures to reshape isoform composition, even when individual junction-level signals appear weak or variable across samples (Fig. 3C). This integrated representation helps reconcile differences between promoter-based, transcript-based, and junction-based analyses and provides a framework for interpreting DIU in the absence of strong gene-level differential expression.

### 3.4 Regulatory Motif Enrichment Implicates Coordinated Transcriptional Control of Promoter Switching

To identify candidate regulatory factors associated with promoter switching and isoform redistribution, we performed motif enrichment analysis on the differentially used promoters, analyzed separately by direction of change. This analysis identified 179 enriched motifs corresponding to 138 transcription factors (Fig. 3D; **Supplementary Table 3**). Among promoters with increased usage in AD, the most significantly enriched motifs corresponded to interferon regulatory factors IRF9 (*p* = 3.6 x 10^−4^), IRF8 (*p* = 6.9 x 10^−4^), and IRF7 (*p* = 1.6 x 10^−3^), together with the STAT2::STAT1 heterodimer motif (*p* = 1.6 x 10^3^), consistent with the type-I interferon programs implicated in AD microglia[14, 15]. Among promoters with decreased usage in AD, the most significantly enriched motifs corresponded to the NF- *κ* B subunits NF- *κ* B2 (*p* = 4.2 x 10^−4^) and NF- *κ* B1 (*p* = 2.4 x 10^−3^)^16^. Twenty-one TFs were enriched at both increased- and decreased-usage promoters, indicating that motif enrichment was not uniformly directional. To prioritize motifs with potential relevance to AD, enriched motifs were mapped to their associated TFs and intersected with a curated AD-related TF set from Hill and Gammie[41], yielding 24 overlapping TFs. These TFs were then linked to nearby differentially used promoters to construct a putative TF-promoter regulatory network (Fig. 3E).

The resulting network showed bidirectional patterns of promoter usage, with TF-associated promoters exhibiting both increased and decreased usage in AD (**Supplementary Fig. 3A**). These findings suggest that promoter switching in AD reflects coordinated transcriptional reprogramming rather than a uniform shift toward promoter activation or repression. To further characterize the regulatory landscape, the top 10 TFs/motifs located in proximity to differentially used promoters are shown in **Supplementary Fig. 3B**.

Together, the motif enrichment and TF-promoter network analyses indicate that the isoform-level changes observed in AD are associated with shared regulatory features across multiple loci. Several implicated TFs are linked to inflammatory, developmental, and cellular stress-response pathways, consistent with molecular processes commonly implicated in AD pathology.

### 3.5 Event-level PSI Analysis Reveals Transcript-structure Features Underlying DIU Genes

To identify the transcript-structure events contributing to AD-associated isoform remodeling, we performed a targeted event-level PSI analysis restricted to transcripts belonging to the 86 genes with significant differential isoform usage. This analysis was designed to decompose the DIU signal into interpretable event classes rather than to serve as an independent genome-wide alternative splicing screen. PSI values were quantified for skipped exon (SE), mutually exclusive exon (MX), retained intron (RI), alternative 5’ splice site (A5), alternative 3’ splice site (A3), alternative first exon (AF), and alternative last exon (AL) events (Fig. 4A), followed by beta regression testing of AD-vs-CT differences with sex included as a covariate.

Across DIU genes, we identified 14 significant differential PSI events at FDR < 0.05 and ∣ΔPSI∣≥0.10 using the additive model (~ condition + sex) (**Supplementary Table 4**). We next tested whether AD-associated splicing changes differed by sex by fitting beta regression models that included a condition-by-sex interaction term. This exploratory analysis identified 14 significant events with evidence of sex-specific splicing effects (Fig. 4B-C). Representative examples included events in *KDSR*, *CXXC1*, and *C11orf80*. A mutually exclusive exon event in *KDSR*, which encodes the 3-ketodihydrosphingosine reductase of the de novo sphingolipid synthesis pathway, showed higher exon inclusion in male CT samples than in female CT samples, with male AD samples showing complete exclusion of the exon (PSI = 0). In *CXXC1*, which encodes the CpG island binding protein CFP1 of the SETD1 H3K4 methyltransferase complex, a retained intron event showed higher PSI values in females than in male AD samples. In *C11orf80*, an alternative first exon event showed sex-dependent usage within AD samples, with inclusion in female and exclusion in male AD samples. Together, these events indicate that sex-associated splicing effects in AD varied in direction across genes and event classes.

Separately, in the additive model, four of the 14 significant events (two RI, one A5, and one A3) fell within a ~ 1.5 kb region of *PLSCR3*, which encodes a mitochondrial phospholipid scramblase involved in cardiolipin distribution and apoptotic signaling. All three shifted in the same direction, with reduced inclusion in AD, indicating a coordinated change at a single locus rather than four independent splicing events.

### 3.6 Partial Concordance with Independent Short-read RNA-seq

We next compared AD-vs-CT transcript-level effect sizes between the ONT long-read dataset and an independent short-read RNA-seq dataset. Across 1,455 shared transcripts, global concordance of AD-associated effect sizes was weak, with Spearman and Pearson correlations of 0.044 and 0.048, respectively. However, among transcripts showing large AD-vs-CT differences in both platforms, 16 of 26 transcripts changed in the same direction, corresponding to 61.5% directional concordance. Applying an additional effect-size similarity filter identified 13 transcripts with large, directionally concordant, and similar AD-vs-CT differences across both long-read and short-read datasets (**Supplementary Fig. 4; Supplementary Table 5**). These results suggest that while short-read RNA-seq does not broadly recapitulate long-read isoform-level effect sizes, a subset of strongly altered transcripts shows reproducible AD-associated expression patterns across independent sequencing platforms.

## 4. DISCUSSION

In this study, we used long-read RNA-seq to characterize transcript isoform remodeling in the AD frontal cortex. By integrating transcript-level quantification, differential isoform usage analysis, promoter usage analysis, targeted alternative splicing event-level analysis, motif enrichment, and comparison with an independent short-read RNA-seq dataset, we found evidence that AD-associated transcriptomic changes extend beyond gene-level expression differences. A central finding of this study is that altered transcription initiation and alternative promoter usage contribute to a subset of isoform diversity in AD, suggesting that promoter-level regulation represents an important layer of transcriptomic dysregulation in diseased brain tissue.

Although gene-level expression analyses are commonly used to define AD-associated transcriptomic changes, they can obscure disease-relevant shifts in transcript structure and isoform composition. Because gene-level counts aggregate all isoforms from a locus, they cannot distinguish whether increased expression reflects greater production of a protein-coding transcript or preferential expression of an isoform predicted to undergo NMD or encode a nonfunctional product. Our differential isoform usage analysis addressed this limitation by identifying genes in which relative isoform usage was redistributed between AD and control samples. These switches included isoforms with predicted functional consequences, including altered NMD sensitivity, highlighting transcript-level changes that would not be resolved by gene-level analysis alone. Notably, NMD-associated isoform switches occurred in both directions, with NMD-sensitive transcripts showing increased usage in either AD or control samples depending on the gene. Together, these findings underscore the importance of transcript-resolved analyses for distinguishing quantitative changes in gene expression from qualitative changes in isoform structure and predicted protein-coding potential.

Importantly, several of the 86 genes with significant differential isoform usage have prior links to AD-relevant biology, supporting the biological relevance of the isoform-switching signal. These include genes involved in synaptic structure and AD-linked proteinopathy, such as CLSTN3[34, 35] and SNCA[37, 38]; proteostasis, autophagy, and ubiquitin-proteasome regulation, including USP10[42, 43], PSMC6[44], and BECN1[45]; inflammatory and microglial or perivascular signaling, including SPP1[46]; and vascular, signaling, or noncoding regulatory pathways, including NOTCH4[39, 40], PVT1[47, 48], COL4A2[49], and EGFR[50-52]. Prior studies have implicated CLSTN3[34, 35] in dystrophic neurite formation around amyloid- *β* plaques, USP10[42, 43] in tau stabilization and aggregation, SPP1[46] in perivascular cell-microglia signaling and synaptic engulfment in AD models, and BECN1[45] in autophagy-mediated amyloid clearance. Similarly, SNCA[37, 38] has been linked to *α* -synuclein co-pathology in AD, PSMC6[44] to proteasome alterations associated with AD progression, NOTCH4[39, 40] to dementia-associated genetic variation, and EGFR[50-52] to amyloid- *β* -induced memory impairment and AD-relevant signaling. Rather than identifying an entirely novel set of AD-unrelated genes, our analysis suggests that known AD-associated pathways may also be remodeled at the isoform level. This finding highlights the value of transcript-resolved analysis for revealing how disease-relevant genes may be altered not only in abundance, but also in transcript structure, promoter usage, and predicted functional output.

Promoter usage analysis suggested that altered TSS selection contributes to a subset of AD-associated isoform changes. Among genes with significant differential isoform usage, 11 exhibited promoter switching, defined by reciprocal differential usage of at least two promoters within the same gene. *NSD2* is a chromatin-modifying enzyme and has not previously been implicated in AD, but a shift in the promoter usage of this gene could in principle propagate to downstream transcriptional programs. At *NSD2*, the promoter favored in AD lies approximately 35 kb downstream of the alternative promoter and initiates a transcript encoding an N-terminally truncated protein product lacking the catalytic SET domain, indicating that promoter switching at this locus alters the domain composition of the encoded chromatin-modifying enzyme rather than only its abundance. This finding indicates that promoter-level redistribution can accompany isoform remodeling in AD, although it does not account for all observed DIU events.

The targeted event-level PSI analysis further showed that DIU genes were associated with multiple transcript-structure event classes (SE, AF, RI, A5, A3, MX, and AL). Two further genes converge on lipid and membrane biology: *KDSR* catalyzes an early step in de novo sphingolipid synthesis, and *PLSCR3* encodes a mitochondrial phospholipid scramblase implicated in cardiolipin distribution and apoptotic signaling. Lipid metabolism and membrane homeostasis are among the processes most consistently implicated in AD genetics and neuropathology, and altered sphingolipid and cardiolipin composition have been reported in AD brain[53]. We therefore regard these events as nominating lipid handling genes for isoform-level follow-up rather than as evidence of gene-specific involvement. *CXXC1* and *C11orf80* have no established relationship to AD, and we do not attribute pathological significance to them here.

*PLSCR3* was distinctive in that four of the 14 significant events mapped to a single ~ 1.5 kb region, with reduced inclusion in AD across all four events. Because SUPPA2 defines overlapping event classes from the same transcripts, these are not independent tests and should be read as one coordinated change at the locus. That several event classes describe the same shift does, however, make it less likely to reflect a single mis-assigned transcript, and identifies *PLSCR3* as the clearest locus-level splicing change in this dataset.

Motif enrichment analysis of differentially used promoters revealed a large set of enriched TF binding motifs, including several factors previously implicated in AD or neuroinflammatory processes. By intersecting enriched motifs with an independently curated AD-related TF set, we identified a focused subset of regulators whose motif presence was associated with directional changes in promoter usage. Notably, TFs exhibited context-dependent effects, being associated with both increased and decreased promoter usage across different genes. This bidirectional behavior is consistent with the known context-specific activity of TFs, which can act as activators or repressors depending on cofactors, chromatin state, and cellular environment[54, 55]. These findings suggest that promoter switching in AD may reflect coordinated but context-dependent transcriptional regulation across multiple loci.

Comparison with an independent short-read RNA-seq dataset provided orthogonal support for a subset of transcript-level AD-associated changes, while also underscoring the inherent limitations of short-read RNA-seq data for isoform-resolved analysis. Across shared transcripts, global concordance of AD-vs-CT effect sizes between platforms was weak, indicating that short-read RNA-seq does not broadly recapitulate isoform-level changes detected by long-read sequencing. However, among transcripts showing large AD-vs-CT differences in both platforms, a majority changed in the same direction, and a smaller subset showed large, directionally concordant, and similar-magnitude effects across datasets. These results suggest that a subset of transcript-level changes identified by long-read RNA-seq is reproducible in an independent short-read RNA-seq cohort, underscoring the value of long-read sequencing for resolving full-length transcript structures, promoter-linked isoforms, and complex patterns of isoform usage.

Several limitations should be considered when interpreting these findings. First, the use of bulk brain tissue limits the ability to attribute promoter and isoform changes to specific cell types. AD frontal cortex contains a mixture of neuronal, glial, vascular, and inflammatory cell populations, and disease-associated shifts in cell composition may contribute to some observed transcriptomic differences. Future studies combining long-read sequencing with single-cell or single-nucleus approaches will be important for resolving cell type-specific promoter usage and isoform regulation. Second, motif enrichment identifies candidate regulatory factors but does not establish direct TF binding or causal regulatory activity. Functional validation will be required to determine whether the implicated TFs directly regulate promoter choice and isoform expression in AD-relevant cellular contexts. Third, the sample size of the long-read dataset was modest, and the short-read comparison should be interpreted as an orthogonal concordance analysis rather than direct validation of full-length isoform models.

Despite these limitations, our study provides a comprehensive view of isoform regulation in the AD frontal cortex and highlights transcription initiation as one regulatory layer shaping transcriptomic diversity in neurodegeneration. By demonstrating that promoter switching occurs in a subset of genes with AD-associated differential isoform usage, and that DIU genes also contain multiple classes of differential PSI events, this work expands the analysis of AD transcriptomic dysregulation beyond gene-level abundance and internal exon choice alone. These findings support a framework in which promoter usage, transcript structure, and splicing jointly contribute to the molecular remodeling of the AD brain.

In conclusion, our analysis reveals that transcriptome remodeling in AD occurs across multiple regulatory layers, including differential isoform usage, promoter switching, and transcriptional regulation. At the RNA level, we identified genes with significant shifts in isoform proportions, including genes with prior links to AD-relevant biology. Promoter usage analysis further identified a subset of DIU genes with altered transcription start site selection, suggesting that promoter switching contributes to isoform remodeling in AD frontal cortex. At the regulatory level, TF motif enrichment analysis of switching promoters implicates key upstream regulators potentially driving these changes. Together, these findings highlight the coordinated impact of transcriptional and post-transcriptional regulation in shaping the molecular landscape of AD.

## Supplementary Material

This is a list of supplementary files associated with this preprint. Click to download.

- SuppMaterials.docx
- SuppMaterials.docx
- SuppMaterials.docx
- SuppMaterials.docx
- SupplementaryFigure14.pdf
- SupplementaryFigure14.pdf
- SupplementaryFigure14.pdf
- SupplementaryFigure14.pdf
- SupplementaryTables.xlsx
- SupplementaryTables.xlsx
- SupplementaryTables.xlsx
- SupplementaryTables.xlsx

## Figures and Tables

**Figure 1:**
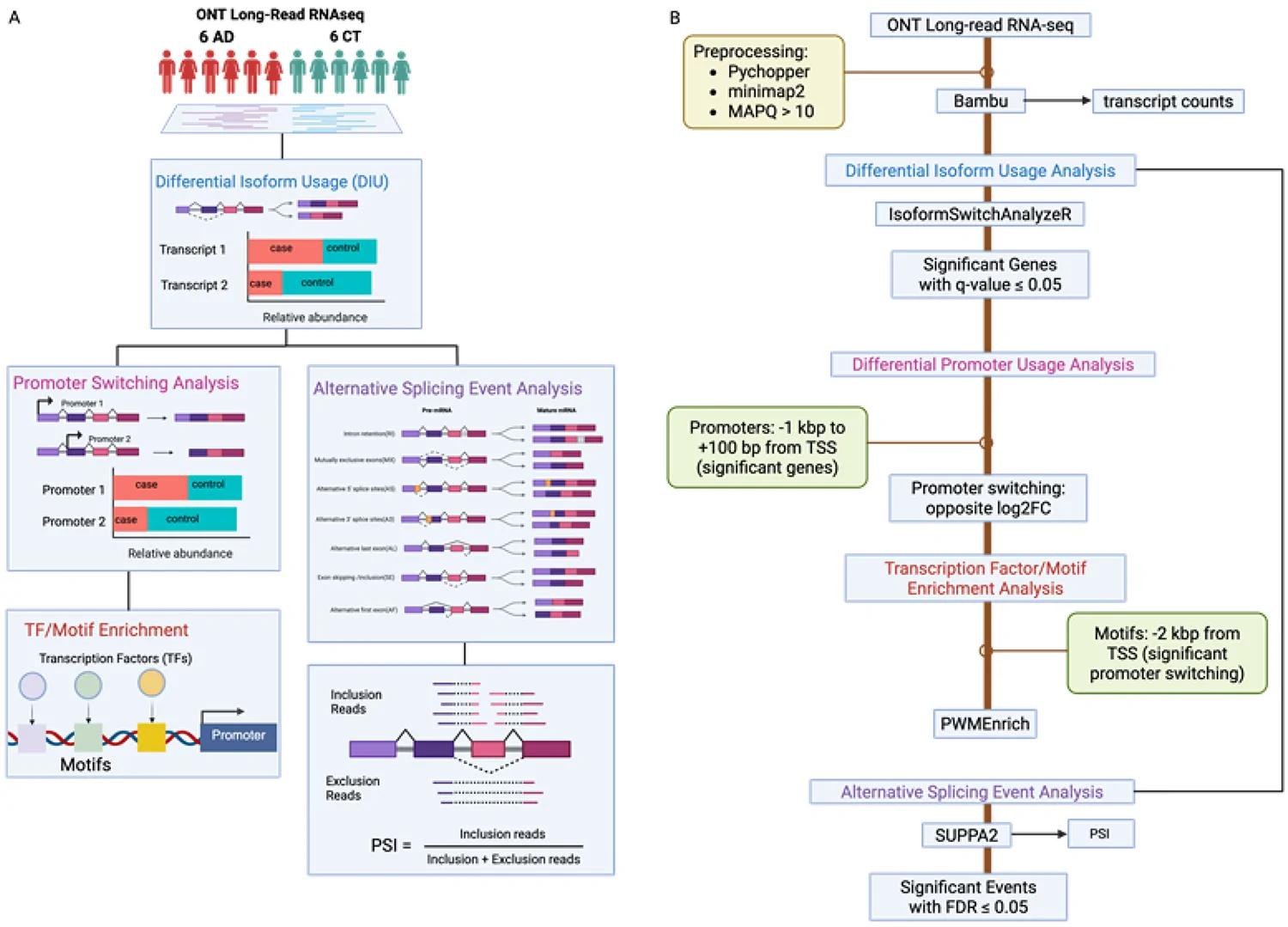
Study design and analytical workflow. Created with BioRender. **(A)** Post-mortem frontal cortex samples from individuals with Alzheimer’s disease (AD; n = 6) and cognitively normal controls (CT; n = 6) were profiled using Oxford Nanopore Technologies (ONT) long-read RNA sequencing. Long-read transcript models were used for differential isoform usage analysis to characterize transcript composition changes, differential promoter usage analysis to identify shifts in transcription start site (TSS) selection, and transcription factor (TF) motif enrichment analyses to infer regulatory mechanisms associated with altered transcript expression. In parallel, differential alternative splicing event analysis based on percent spliced in (PSI) values was performed to identify event-level splicing alterations between AD and CT samples. **(B)**Bioinformatics workflow for long-read RNA-seq processing and downstream analyses, including transcriptome assembly, transcript quantification, isoform and promoter usage analyses, alternative splicing event detection, and functional enrichment analyses. (Icons used in this figure were available to the authors through an institutional license to biorender.com.)

**Figure 2:**
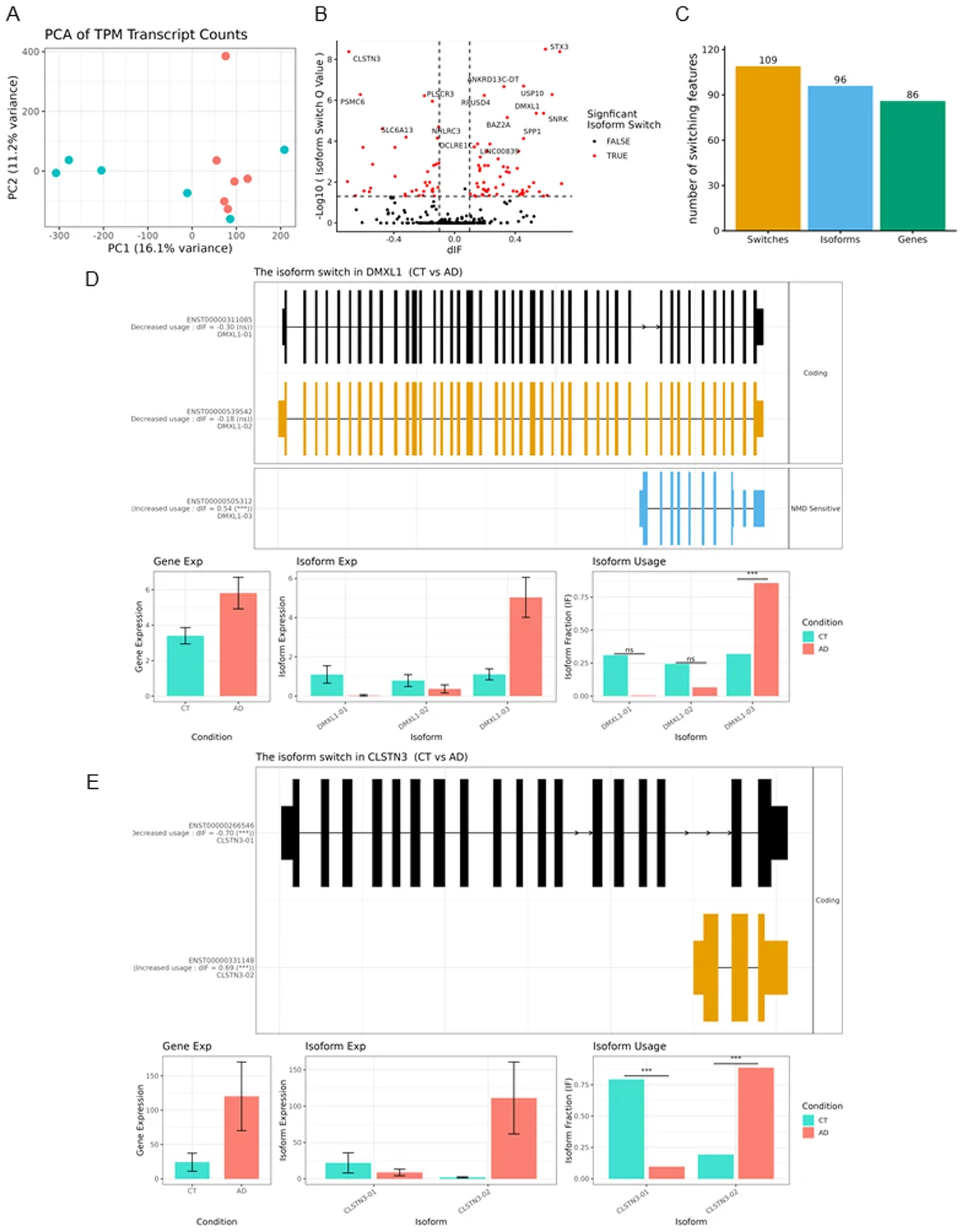
Long-read differential isoform usage analysis. **(A)**PCA of transcript TPM values between 6 AD (salmon points) and 6 CT (turquoise points). **(B)** Volcano plot illustrating isoform switching based on differential isoform fraction (dIF) and statistical significance. Isoforms meeting the significance criteria of ∣dIF∣ > 10% and FDR < 0.05 are highlighted in red. **(C)** Bar plot summarizing the number of genes and isoforms with significant isoform-switching events. **(D–E)** Isoform switch plots of DMXL1 **(D)** and CLSTN3 **(E)** that were significant for DIU. The top panel represents the transcript structure of the gene, annotating which transcript had increased or decreased usage and its differential Isoform Fraction (dIF) value in AD. Bottom panels represent the gene expression (left), isoform expression (center), and isoform usage (right) values comparing AD vs CT. AD bars are salmon, and CT bars are turquoise. The DEXSeq statistical test result is annotated on the bars (ns: not significant; *: p < 0.05, **: p < 0.01, ***: p < 0.001).

**Figure 3:**
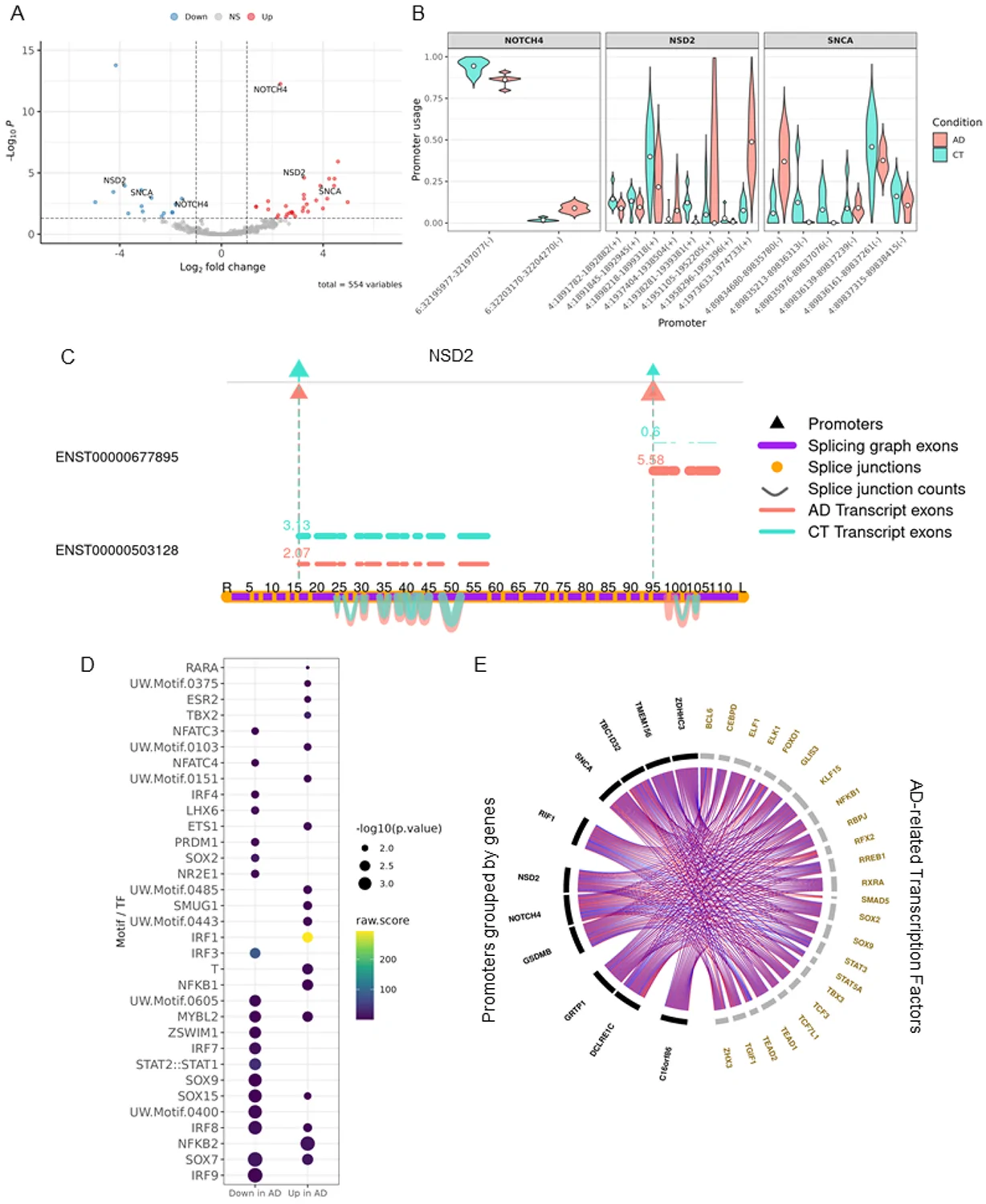
Long-read promoter switching analysis and TF/motif enrichment analysis. **(A)** Volcano plot showing significant (adjusted *p* 0.05) promoters differentially used, with red dots as the upregulated promoters (log_2_ (fold change) > 1) and blue dots as the downregulated promoters (log_2_ (fold change) < −1). The promoters of three AD-related genes (*NOTCH4*, *SNCA*, and *NSD2*) are labeled on both the up- and downregulated sections to display promoter switching. **(B)** Violin plots of the three genes labeled in A. The x-axis shows the individual promoters of the gene, and the y-axis shows their promoter usage between the AD and CT individuals. The white dot represents the mean promoter usage expression. **(C)** Splicing graph representation of *NSD2* integrating promoter, transcript, and splice junction counts. The yellow nodes denote splice junctions, and purple edges represent exonic segments spanning the *NSD2* gene locus. The two annotated NSD2 transcripts are shown as linear paths through the graph, with line thickness proportional to transcript abundance. Promoter usage is indicated by triangular nodes, with triangle size reflecting promoter-level counts. Splice junction usage is shown as curved arcs, with arc thickness proportional to junction counts. For all count types, turquoise indicated mean values across controls, and salmon indicated mean values across AD. **(D)** Dot plot showing the top TFs that are associated with the promoters with decreased and increased usage, with the size of the dot corresponding to their significance (−log10(p-value)), and the colors of the dots are the raw score, which reflects the motif match strength. **(E)** Circos plot showing TFs/motifs associations with genes undergoing promoter switching in AD. Hierarchical clustering (k = 4) was performed to create the gaps between the genes. Links connect TFs to genes whose promoters contain corresponding binding motifs. Red links indicate promoters with increased usage in AD, while blue links indicate promoters with decreased usage. Link width reflects motif match strength.

**Figure 4:**
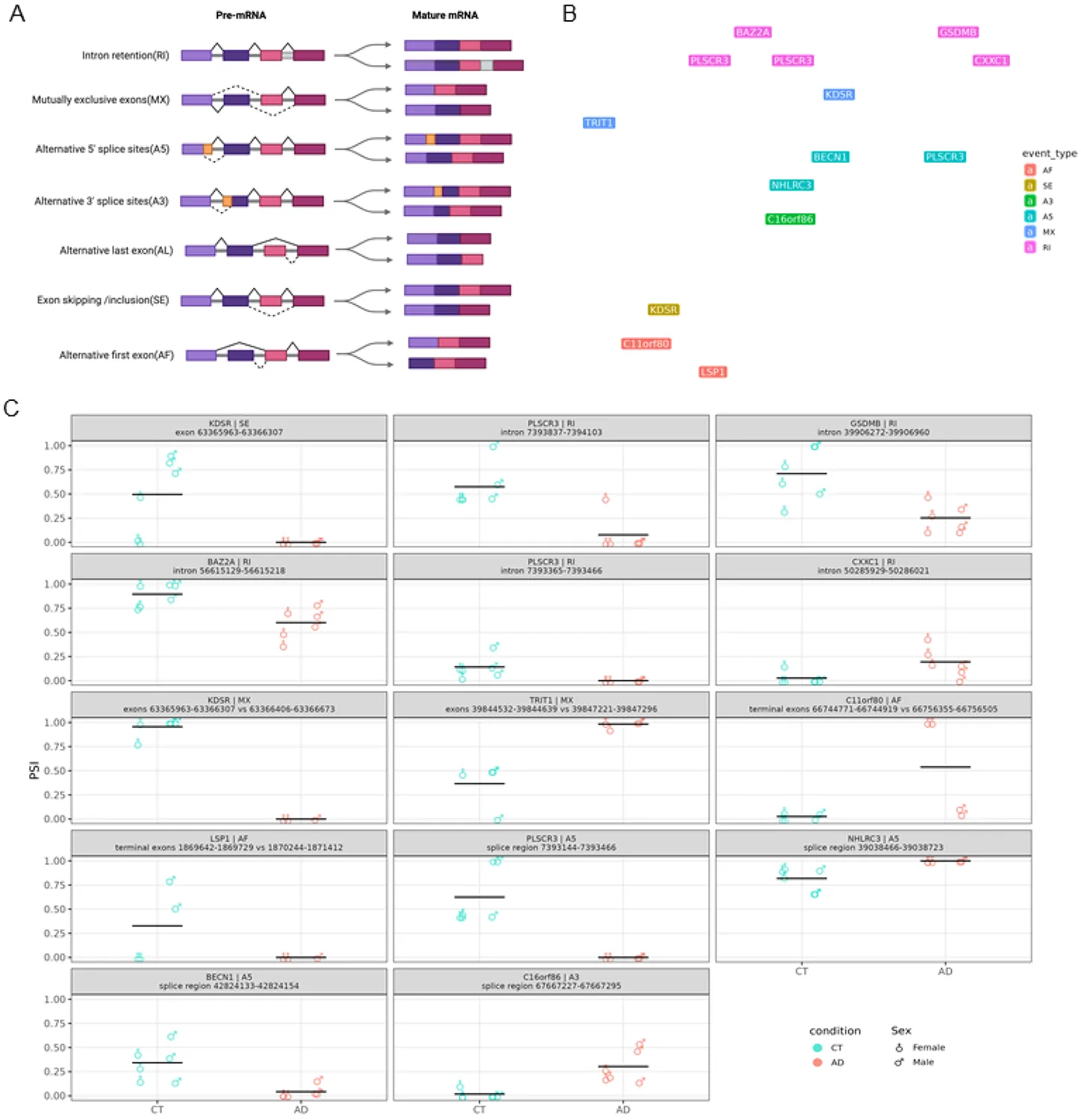
Event-level alternative splicing analysis. **(A)** Created with BioRender. Diagram showing the different alternative splicing events detected by SUPPA2. **(B)** Gene cloud showing genes associated with alternative splicing events. Each rectangle represents a gene, labeled by its gene symbol, and is colored according to the alternative splicing event type. **(C)** Alternative splicing events exhibiting a significant sex-by-condition interaction in the beta regression analysis (model: PSI ~ condition + sex + condition:sex). Each facet displays the PSI values for one significant splicing event in control (CT) and Alzheimer’s disease (AD) samples. Individual points represent samples with symbols denoting sex. Horizontal bars indicate the group mean PSI. Panel headers list the gene, splicing event type, and the genomic coordinates of the affected exon(s). (Icons used in this figure were available to the authors through an institutional license to biorender.com.)

## Data Availability

All datasets analyzed in this study are publicly available. The Oxford Nanopore long-read RNA-sequencing data are available through Synapse (syn52047893) and the Sequence Read Archive (SRP456327). The independent short-read RNA-sequencing dataset is available through the European Nucleotide Archive (ERP161086; PRJEB76583). No new primary sequencing data were generated in this study.

## Code availability

All code used in the manuscript is publicly available at GitHub (https://github.com/jelardaquino/TSSwitch) or upon request to the authors.
